# Comparison of Root Ecological Stoichiometry between Non-Growing Season and Growing Season of Grassland on the Chang Tang Plateau

**DOI:** 10.3390/ijerph192214628

**Published:** 2022-11-08

**Authors:** Xingxing Ma, Yan Yan, Jiangtao Hong, Xiaodan Wang

**Affiliations:** 1 School of Geographical Sciences, Shanxi Normal University, Taiyuan 030031, China; 2 Institute of Mountain Hazards and Environment, Chinese Academy of Sciences, Chengdu 610041, China

**Keywords:** root system, ecological stoichiometry, alpine grassland, soil nutrients

## Abstract

Root C: N: P stoichiometry affect the geochemical cycles of ecosystems, while a few studies were conducted on it and its relationship with soil nutrients, especially in the non-growing season. In this study, we investigated the root C:N:P stoichiometry of alpine steppe(AS), alpine meadow steppe(AMS), and alpine meadow(AM) in April (non-growing season) and August(growing season) in 2013. The results showed that: (1) There were no differences in root C, N, P, C: N, C:P, and N:P with a depth of AS in April. However, root C and C: N increased with depth, while N and N:P decreased with a depth of AS in August. In both months, the variation of root C, N, P, C: N with depth in AM was consistent with that of AS in August, and root C at deep layer decreased in August, which indicated roots of AM began to grow in April No significant difference of root C, N, C: N and N:P with depth was found, while P and C:P varied between the two months of AMS. Root P content at 20–30 cm was higher than that of other soil layers in April, which was significantly higher than that of AS, while no significant difference was found in August. (2) Grassland types had significant effects on soil nutrients (except TP) at 0–10 cm and 20–30 cm soil layers. (3) No significant correlation between soil nutrients and root C, N, P, C: N, C: P, and N: P was found in April. Soil TN and AN content had a significant positive correlation with root N: P, indicating that soil nitrogen was the primary N source of the root. Soil TP and AP were significantly negatively correlated with root C and C: N in August. This study can provide basic data and provide theoretical support for further understanding the role of grassland roots in nutrient cycling.

## 1. Introduction

Carbon(C), nitrogen(N), and phosphorus(P) are essential elements for plants, which play different functions and roles in plant growth and development. C is a structural element in plants, and the content of N and P is affected by environmental factors and biological factors. N and P are key factors that limit the primary productivity of terrestrial ecosystems [1], which affect the species richness and community composition of ecosystems, especially in alpine and polar ecosystems that are limited by low temperatures [2]. C: N and C: P are important for determining the photosynthetic carbon sequestration capacity of leaves [3]. N: P can indicate nutrient supply status of soil to plants, and is widely used to determine the limiting pattern of N and P nutrients in plant-soil systems [4]. C, N, P contents and their ratio of plants affect the nutrient cycle of the plant-soil system, and C, N, P stoichiometric characteristics of different components from the ecosystem play an important role in ecosystem structure and function maintenance [5]. Therefore, it is of great importance to study the alpine plants’ C, N, and P ecological stoichiometry.

The root is an important part of alpine plants, which can fix plants and absorb and transport water and nutrients needed for plant growth and development [6].The decomposition of plant roots, leaching, and release of root nutrients are important links of nutrient migration [7]. Many studies have been carried out on root biomass and its vertical distribution of alpine plants in the growing season [8,9]. Stoichiometry of C: N: P has been applied to understand ecosystem nutrients limitation, community dynamism [10], nutrient use efficiency [11], and the global biogeochemical cycle [2]. Studies on plant N and P are focused on the aboveground part [12,13], such as leaves’ N and P contents and ecological stoichiometric under the effects of the enclosure, grazing, and N deposition [14,15], while studies on root ecological stoichiometric are still a little insufficient. Soil C: N:P ratios can be used to assess soil nutrient status, control plant growth, and demonstrate plant nutritional conditions [4]. Its relationship with plants is thought to show how plants use resources in an environment that is always changing [16]. Opposite relationship of C:N:P stoichiometry in the leaves and roots with rhizosphere soil was found in subtropical plantations [17]. The responses of N:P stoichiometry to soil P differed among functional groups [17]. 

Tibet is one of the important grasslands in China, in which the grassland area accounts for about 21% of the natural grassland area in China [18]. Alpine steppe (AS), alpine meadow steppe (AMS), and alpine meadow (AM) are the most important grassland types in Tibet [19]. Alpine plants have evolved special adaptations to cope with cold and dry conditions, and the root biomass of the grassland ecosystem is about 2–5 times that of the aboveground biomass [20,21]. In this study, we took roots of AS, AMS, and AM located in Xainza County in Nagqu prefecture as the research object, and determined root C, N, P content and soil nutrients content. Our objectives were: (1) to find out if there could be differences of root C, N, and P ecological stoichiometry features of the alpine grassland in different periods, (2) to patterns of soil nutrients, and (3) relationships between soil nutrients content and root C, N, P, C:N, C:P, N:P. 

## 2. Materials and Methods

### 2.1. Experimental Design

The experimental sites were located near Xainza Alpine Grassland and Wetland Ecosystem Observation Station (30°57′ N, 88°42′ E, 4675 m) in Xainza County, Tibet Autonomous Region. The region is a semi-arid monsoon climate of plateau sub-frigid zone, cold and dry, with thin air and strong sunshine radiation. The average annual temperature and annual precipitation are 0 °C and 300 mm, with most of the precipitation occurring from May to September. The duration of the frost period was 279.1 days, and the annual sunshine duration was 2915.5 h. The vegetation in this area is mainly AS, and there are also AMS and AM in a certain areas. The AS is dominated by *Stipa purpurea* and *Carex moocroftii*, associated with *Leontopodium nanum*, *Oxytropis*, *Stellera chamaejasme*, etc. The AMS is mainly composed of *Carex Moocroftii* and *Kobresia humilis*. The AM is dominated by *Kobresia humilis* and *Kobresia macrantha*, with some miscellaneous grasses. The soil types of AS and AM are: low-temperature arid soil and frozen primordial soil in order [22]. The soil of AMS is between the alpine steppe and alpine meadow.

### 2.2. Sample Collection and Analysis

The AS site is located in the Xainza Alpine Grassland and Wetland Observation Station, while the AMS and AM sample sites are located outside the wetland station. The AS, AMS, and AM are distributed from east to west in a row, with an interval of 2 km. Xainza river and Kering Tso are distributed in the west of the AM. Root samples were collected in April and August of 2013, with three replicates for each grassland. The root samples were excavated with soil blocks (50 cm square and 30 cm in depth), with every 10 cm as a layer. The roots obtained were divided into dead roots and live roots according to color and elasticity. The dead roots were removed and the live roots were put into cloth bags and brought back to the laboratory. After the root samples were washed with clean water, they were put into the envelopes and oven dried at 65 °C to a constant weight. The dried root samples were ground into a fine powder, which could pass through a 0.25 mm sieve, and then bagged for later use. Soil samples of each layer (0–10 cm, 10–20 cm, and 20–30 cm) were collected at the same time in August, and air-dried after root removal. The contents of root C, root N, soil total C (TC), and soil total N (TN) were determined by an elemental analyzer (Vario Macro Cube, Elementar, Germany), and the contents of root P and soil total P (TP) were determined by phosphorus molybdate blue spectrophotometry. The soil available P (AP) and soil alkali-hydrolyzed nitrogen (AN) were adopted by the molybdenum blue method and alkali-hydrolyzed diffusion method. All the data were expressed in terms of mass. The C: N (ratio of [C] and [N]), C: P (ratio of [C] and [P]), and N: P (ratio of [N] and [P]) ratios were also expressed on a mass basis.

### 2.3. Statistical Analyses

The differences in plant C: N:P stoichiometry among the different grassland types and soil depths were processed by one-way ANOVA. The relationships between soil nutrients and root C: N:P stoichiometry was analyzed by regression analysis. The differences in root C: N:P stoichiometry between April and August were processed using a *t*-test. The statistical analyses were performed using SPSS.17.0 (SPSS Inc., Chicago, IL, USA), and the statistical data were plotted using SigmaPlot.12.5 (Systat Software, Richmond, CA, USA).

## 3. Results

### 3.1. Contents and Ratios of C, N and P in Roots in April

**Figure 1:**
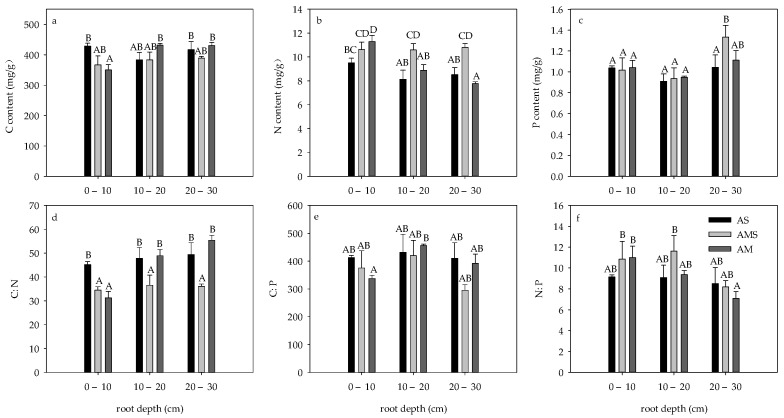
Root carbon, nitrogen, phosphorus, C:N, C:P, and N:P in April. (**a**) Root C content; (**b**) Root N content; (**c**) Root P content; (**d**) Root C:N; (**e**) Root C:P; (**f**) Root N:P. Different letters correspond to statistical differences at 0.05 level.

C and N contents in roots of AS and AMS showed no significant difference with the change of depth, while the C content in 0–10 cm roots of AM was significantly lower than that in other soil layers, while the N content was significantly higher than that in other soil layers (*p* < 0.05, Figure 1a,b). The root C content of 0–10 cm in AS was significantly higher than that in AM, but there was no significant difference in the root C content of the three grasslands in other soil layers (*p* > 0.05, Figure 1a,b). The root N content in 0–10 cm AS was significantly lower than that in AM, and the root N content in AMS in other soil layers was significantly higher than that in the other two grasslands (*p* < 0.05, Figure 1b). There was no significant difference in root P content between AS and AM with the change of depth, while the root P content in 20–30 cm of AMS was significantly higher than that in other soil layers, and the root P content in 20–30 cm was significantly higher than that in AS (*p* < 0.05, Figure 1c).

The root C:N, C:P, and N:P of AS and AMS showed no significant difference with depth, while the root C:N and C:P of 0–10 cm were the lowest and the root N:P was the highest in AM. The C:N of roots of 0–10 cm AM and AMS at all depths was lower than that of other roots (*p* < 0.05, Figure 1d). There was no significant difference in root C:P and N:P at the same depth among the three grasslands (*p* > 0.05, Figure 1e,f).

### 3.2. Contents and Ratios of C, N and P in Roots in August

**Figure 2:**
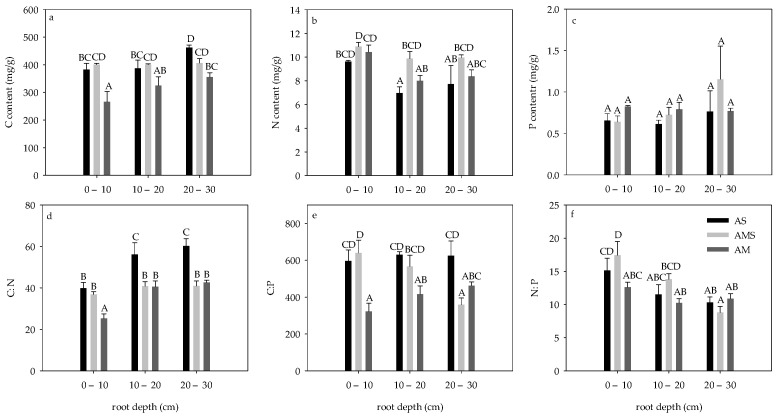
Root carbon, nitrogen, phosphorus, C: N, C: P, and N: P in August. (**a**) Root C content; (**b**) Root N content; (**c**) Root P content; (**d**) Root C: N; (**e**) Root C:P; (**f**) Root N:P. Root carbon content. Different letters correspond to statistical differences at 0.05 level.

Root C content of both AS and AM was the lowest in 0–10 cm and the highest in 20–30 cm, while root C content of AMS showed no significant difference with depth change. Root C content of all layers of AM was lower than that of other grasslands (Figure 2a). Root N content of both AS and AM was the highest in 0–10 cm. The N content in the roots of AMS showed no significant difference with depth change, and the N content in roots of 10–20 cm in AS was significantly lower than that in AMS (*p* < 0.05, Figure 2b). There was no significant difference in P content in all roots (*p* > 0.05, Figure 2c).

Root C:N in AS and AM at 0–10 cm was the lowest, while it had no significant difference in AMS. The root C:N at the same depth was significantly higher in AS than in AM (*p* < 0.05, Figure 2d). The root C:P of AMS decreased significantly with depth. The root C:P of 0–10 cm and 20–30 cm in AM was the lowest, while that at 20–30 cm in AMS was the lowest (*p* < 0.05, Figure 2e). N:P decreased significantly with depth in AS and AMS, while no significant change with the depth was found in AM, and the root N:P in 0–10 cm AMS was significantly higher than that in the AM at the same depth (*p* < 0.05, Figure 2f).

### 3.3. Difference of Root C:N:P Stoichiometry between April and August

The variations of root C, N, P contents and ratios of all the grassland were not consistent in different months. A decrease in AS root P content at 0–10 and 10–20 cm was found, which led to a significant increase in N:P at 0–10 cm and C:P at 10–20 cm in August (*p* < 0.05, Table 1). All root C, N, and P contents and ratios had no difference between the two months, except that C:P of 0–10 cm in AMS increased (*p* < 0.05, Table 1). In the AM in August, C and P contents and C: N in roots of 10–20 and 20–30 cm and 20–30 cm were significantly decreased, while N:P in roots of 20–30 cm was increased (*p* < 0.05, Table 1).

### 3.4. Patterns of C, N, P, AN and AP Contents of Soil

**Figure 3:**
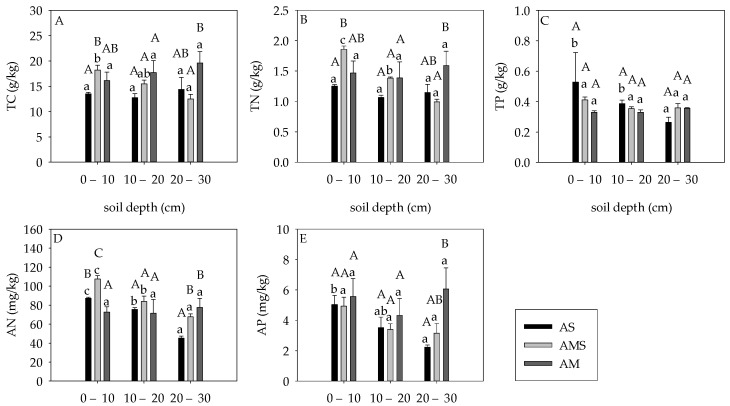
Soil carbon, nitrogen, phosphorus, AN, and AP content at different soil layers. (**A**) Soil TC content; (**B**) Soil TN content; (**C**) Soil TP content; (**D**) Soil AN content; (**E**) Soil AP content. Different capital letters indicate statistical differences at 0.05 level between different grassland types at the same depth. Different lowercase letters represent differences at the 0.05 level between different depths of the same grassland.

Soil depth had no significant effects on all soil nutrient content in AM, which varied in AS and AMS. The soil TC and TN contents at 0–10 cm are higher than that at 20–30 cm in AMS, while soil depth had no significant effects on that of AS (Figure 3A,B). The soil TP and AP contents at 0–10 cm were significantly higher than that in 20–30 cm of AS, and no difference was found in AMS (Figure 3C,E). Soil AN content were in the order of 0–10 cm > 10–20 cm > 20–30 cm in AS and AMS (Figure 3D).

Grassland types had significant effects on soil TC, TN, AN, and AP contents at the same layer (*p* < 0.05, Figure 3A–D), while soil TP showed no significance with grassland types at the same soil layers (*p* > 0.05, Figure 3E). The markable difference of soil nutrients (except TP) mainly concentrated at 0–10 cm and 20–30 cm soil depths, and no significant difference was found between them at 10–20 cm depth. Soil TC content was the highest in AMS (18.20 ± 0.94 g/kg) and the lowest in AS (13.51 ± 0.28 g/kg) at 0–10 cm depth, and it was the highest in AM (19.58 ± 2.24 g/kg) and the lowest in AMS (12.49 ± 0.90 g/kg) at 20–30 cm depth. Soil TN content in AS (1.25 ± 0.03 g/kg) was significantly lower than that in AMS (1.86 ± 0.06 g/kg) at 0–10 cm, while it was markable lower in AMS (0.99 ± 0.04 g/kg) than AM (1.59 ± 0.23 g/kg) at 20–30 cm. Soil AN content in AM was significantly lower than that in AMS at 0–10 cm depth, and it in AM was higher than AS at 20–30 cm depth. Soil AP content in AS (2.23 ± 0.16 mg/kg) was significantly lower than that in AM (6.07 ± 1.38 mg/kg) at 20–30 cm depth, but there was no significant difference among grasslands at the other two soil layers.

### 3.5. Relationship between Root C:N:P Stoichiometry and Soil Nutrients Content

There was no significant correlation between soil nutrients and root C, N, P, C:N, C:P and N:P in April (*p* > 0.05, Table 2), while relationships between soil nutrient content and root C, N, P, C:N, C:P and N:P varied in August. No significant correlation was observed between soil TC content and root C: N: P stoichiometry in the two periods (*p* > 0.05, Table 2). Soil TN content and AN both showed a significant positive correlation with root N:P in August (*p* < 0.05, Table 2), and no significant correlation with other indexes (*p* > 0.05, Table 2). Soil TP and AP were significantly negatively correlated with C and C:N in roots during August (*p* < 0.05, Table 2), which had no significant correlation with other indexes (*p* > 0.05, Table 2). 

## 4. Discussion

Carbon, nitrogen, and phosphorus are important elements in the process of plant growth and development, and their contents and stoichiometric ratios can reflect the efficiency of growth and nutrient supply. The root C content in our study (350.07 ± 37.11–411.92 ± 10.38 mg/g) was basically consistent with the average root C content of the four species in Hulunbuir grassland (382–423 mg/g) [23]. We found that the root N content in our study (8.28 ± 0.43–10.47 ± 0.51 mg/g) was lower than the global root N content (11.1 mg/g) [24]. However, the root P content in our study (0.71 ± 0.01–1.16 ± 0.09 mg/g) was slightly higher than that of global plant roots (0.77 mg/g) [24]. However, the N and P contents were lower than the average N content (14.58–32.91 mg/g) and P content (1.26–2.65 mg/g) of the roots of the four plants in Hulunbuir grassland [23]. A study on the northern grassland transect also confirmed that the root N content of plants in Inner Mongolia grassland was significantly higher than that in the Qinghai-Tibet Plateau [25].

The average root C: N of the three grasslands in our study (34.02 ± 2.19–47.91 ± 1.06) was basically the same as that of the grassland transect on the Qinghai-Tibet Plateau (35.65 ± 12.25) and higher than that of the grassland transect of Inner Mongolia (24.33 ± 10.95) [25]. The average root N:P (7.93 ± 0.64–15.06 ± 0.74) was slightly lower than that of the Tibetan Plateau grassland (21.63 ± 7.13) and the Inner Mongolia grassland (22.67 ± 8.78). The reasons for the difference may be as follows: (1) Different habitat conditions, such as latitude, altitude, and average annual mild precipitation, lead to the difference in element content in roots [26,27,28]. Root N content decreased with increasing altitude, increased with increasing annual temperature, and decreased with increasing annual precipitation. The data of plant root elements in Inner Mongolia and Qinghai-Tibet Plateau are all large-scale transect data, covering a wide range of regions. A study on 19 species of alpine grassland vegetation in Tibet showed that the average root C:N was 29.58 [29]. The maximum value of root C:N in AS was 39.42, and the minimum value of root C:N in the alpine shrub meadow was 27.83 [29]. (2) The N:P of different species varied greatly, and different species compositions affected the N:P of grassland communities. For example, in Hulunbuir grassland, the highest root N:P was in *Thermopsis lanceolata* (17.49), the lowest in *Cymbaria dahurica* (6.62), and the other two plants were *Leymus chinensis Tzvel* (11.53) and *Potentilla acaulis Linn* (11.16) [17]. In addition, it is generally believed that plants with N:P less than 14 will be limited by N element, while those with N:P greater than 16 will be limited by the P element [30]. The average root N:P of the three grassland species in this study was less than 16, while the root N:P of the Inner Mongolia region and the Qinghai-Tibet Plateau region were all greater than 16, indicating that there were differences in limiting elements affecting root growth. C:P (365.72 ± 32.89~537.96 ± 45.19) was lower than that in Inner Mongolia (522.32 ± 196.15) and Qinghai-Tibet Plateau (757.60 ± 343.26) [31].

C maintains good homeostasis in plants [32], and root C decreases with the increase of depth in August in AS, which may be mainly due to the dilution effect [33], that is, root C content maintains a dynamic balance. When biomass increases, the C content will decrease. The root biomass of AM was significantly higher than the others, hence the dilution effect was the strongest. Based on the results, we inferred that roots of AM had begun to grow in April, while the roots of the AS and AMS had not yet begun to grow. After vegetation returns to green, the aboveground part needs more N element to participate in respiration and photosynthesis, and a large amount of N element is transported by roots to stems and leaves and other organs, which is also an important reason for the high content of N in surface roots of AM and AS in August [12,32]. In August, most alpine plants are in the flowering and propagation stage, when a large amount of P element is needed, and it resulted in a significant decrease in root P content.

There was no significant difference in root C, N, C: N, and N:P with depth between the two months of AMS, while the changes of P and C:P were different between the two stages. In April, the root P content at 20–30 cm was significantly higher than that of other soil layers, and significantly higher than that of AS, but no significant difference was observed in August. The C:P at 0–10 cm in AMS increased in August. In April, C:P did not change significantly with depth, while it decreased significantly with depth in August. C:P is affected by both C content and P content. The root C content of AMS at the same depth did not increase significantly in August, and the root P content did not decrease significantly. This is the reason why C:P varies with depth in different months. It was found in previous studies that the distribution ratio of root biomass decreased in the growing season of AMS [20], which may be the reason for the insignificant increase of root C content in the growing season. In addition, plants entered the reproductive stage in August, and more P was transported to the reproductive organs, which was the reason for the significant increase in surface C:P. At the same time, the variation of C content and P content at different depths resulted in a significant decrease of C:P with soil depth.

## 5. Conclusions

Our study examined the nutrient stoichiometry variation law in root C, N, and P content and their ratios of AS, AMS, and AM between two months. The C, N, and P stoichiometries differed significantly between April and August with different grassland types (AS, AMS, and AM) and depth. A decrease of P content in shallow roots was found in AS in August, while a decrease of C and P content in deep roots was found and all root C, N, and P contents and ratios except C:P had no difference between the two months. The difference in changes in root stoichiometry indicate different nutrients nutrient uptake strategies and biomass allocation patterns. Soil TN and AN content had a significant positive correlation with root N: P, indicating that soil nitrogen was the primary N source of the root. Soil TP and AP were significantly negatively correlated with root C and C: N in August, which is because phosphorus is relatively weak in mobility and needs intensive searching by roots to be absorbed. 

## Figures and Tables

**Table 1:** *T* value of root C, N, P, C:N, C:P, and N:P between April and August.

Type	Depth (cm)	Root C	Root N	Root P	Root C:N	Root C:P	Root N:P
AS	0–10	1.884	−0.283	4.520 *	1.741	−3.049	−3.219 *
	10–20	−0.104	1.276	3.507 *	−1.150	−2.965 *	−1.278
	20–30	−1.632	0.476	1.005	−1.775	−2.202	−1.035
AMS	0–10	−1.138	−0.371	2.733	−1.256	−2.843 *	−2.442
	10–20	−0.666	0.900	1.543	−0.901	−1.806	−1.286
	20–30	−1.049	2.104	0.428	−1.842	−1.520	−0.567
AM	0–10	2.051	1.095	3.112	1.700	0.321	−1.225
	10–20	3.307 *	1.359	1.846	2.167	0.861	−1.180
	20–30	4.017 *	−1.058	3.526 *	5.411 **	−1.829	−3.923 *

“*” corresponds to statistical differences at 0.05 level. “**” corresponds to statistical differences at 0.01 level.

**Table 2:** Standardized Coefficients between soil nutrients and root C, N, P, C:N, C:P, and N:P.

	Month	Root C	Root N	Root P	Root C:N	Root C:P	Root N:P
Soil TC	April	0.112	−0.133	−0.160	0.147	0.178	0.012
	August	−0.197	0.162	−0.055	−0.226	−0.098	0.129
Soil TN	April	−0.082	0.045	−0.212	−0.068	0.102	0.160
	August	−0.174	0.289	−0.084	−0.311	0.065	0.400 *
Soil TP	April	−0.362	0.310	−0.072	−0.357	−0.134	0.254
	August	−0.561 **	0.186	−0.057	−0.486 *	−0.315	0.107
Soil AN	April	−0.233	0.197	−0.058	−0.256	−0.074	0.187
	August	−0.086	0.374	−0.219	−0.357	0.247	0.618 **
Soil AP	April	−0.139	−0.003	0.024	−0.047	−0.142	−0.038
	August	−0.489 *	0.146	−0.025	−0.440 *	−0.151	0.262

“*” corresponds to statistical differences at 0.05 level. “**” corresponds to statistical differences at 0.01 level.

## Data Availability

Data can be obtained by contacting the first or corresponding author by email.
